# Social attachments and traumatic stress

**DOI:** 10.3402/ejpt.v7.29065

**Published:** 2016-03-18

**Authors:** Richard A. Bryant

**Affiliations:** School of Psychology, University of New South Wales, Sydney, Australia

**Keywords:** Attachment, trauma, social network, social processes

## Abstract

The extent to which we engage with our social world has been central to our survival as a species and, accordingly, is relevant to how we cope with trauma and adversity. This review summarises current knowledge about the importance of social connections from an evolutionary perspective, as well as integrating this with a discussion of prevailing attachment theories. Experimental research supporting the potential benefit of attachments for managing adversity are presented, along with a review of how these benefits are moderated by individual differences in attachment style. The potential impact of trauma on attachment systems, and the manner in which this can compound trauma stress is discussed. Finally, a broader overview of social network analysis is introduced and it is proposed that a more sociocentric framework of trauma response would promote a fuller understanding of how social processes moderate trauma response.

There is overwhelming evidence that social attachments play a critical role in how humans manage adversity. Accordingly, it is not surprising that social processes may serve a critical function in how people respond to trauma. This review considers from a theoretical perspective how social attachments may impact trauma response, the role of attachment theory, and the need for the trauma field to pay closer attention to social processes in understanding trauma response at both the individual and community levels.

## Attachment as a means to survival

Many theorists have noted that to survive as a species we have had to work together to manage many threats to our species. To deal with the threats of predators in prehistoric times would have required the collective efforts of many individuals—it would have been much easier to ward off a sabre-tooth tiger with the help of 10 others than trying to do it by yourself. Similarly, building a cabin to protect oneself from the elements is much more effective with the assistance of others than trying to achieve the whole endeavour unaided. Neuroscientists have developed some intriguing theories to account for this proposal, including the idea that the human brain developed beyond those of other species to specifically allow us to connect with each other (Lieberman, 2013). For example, humans have relatively small brains compared with larger animals (e.g., elephants or whales). Despite this comparison, when one takes into account the size of the brain required to maintain core bodily functions (which is determined largely by how big the body is), humans have proportionally larger brains than any other species (Roth & Dicke, 2005). It has been argued that the neocortex in humans is proportionally larger and it allows humans to interact with larger social networks. In fact, some neuroscientists argue that the basic wiring of the human brain in its resting state (called the default mode) is actually very similar to neural circuitry that is activated during social cognition (Lieberman, 2013). According to this argument, as a species we have evolved to interact with each other to allow us to thrive in our environment, and this may be reflected in fundamental neural circuitry that drives our everyday functioning. Specifically, it is proposed that humans have developed this capacity to interact in such an intertwined way because they rely on each other to survive and defend against threats, obtain necessary resources to thrive, and collaborate to manage the environment (Fitzsimons, Finkel, & vanDellen, 2015).

## Attachment theory

The notion that how we relate to each other is pivotal to our survival and psychological health is not new. Attachment theories posit that humans, as well as many other species, learn from an early age to seek refuge in trusted others in times of need; caregivers provide us with food, nurture, and protection when we are vulnerable. Early research conducted by Harry Harlow found that monkeys repeatedly sought out a replica monkey “mother” that was made of cloth, rather than an alternate replica that was made of wire—even though the latter provided milk and the former did not (Seay & Harlow, 1965). Harlow argued that the baby monkeys sought out the cloth replicas because of the innate need to seek “comfort contact.”

Attachment theories posit that whereas this support is initially provided by primary caregivers, others will assume this role as we age (Mikulincer, Shaver, & Pereg, 2005). Attachment theories, exemplified by John Bowlby (1982), posit that people internalise attachment representations, such that mental representations of attachment figures acquire comparable soothing effects. Bowlby placed strong emphasis on this process, such that to achieve optimal psychological functioning “the infant and the young child should experience a warm, intimate and continuous relationship with his [or her] mother (or permanent mother substitute) in which both find satisfaction and enjoyment” (Bowlby, 1951; p.11). An internal working model of the individual's attachment security is subsequently formed throughout infancy and childhood, and developed primarily according to their caregiver's behaviour.

The development of our attachment systems represents a core emotion regulation strategy because we learn from the cradle to turn to trusted others at times of threat. Consistent with this proposal is much evidence that individuals tend to seek attachment representations when they are presented with real or symbolic threats. For example, unconscious exposure to distressing stimuli increases people's tendency to access the names of attachment figures (Mikulincer, Gillath, & Shaver, 2002). It has also been found that in response to threatening stimuli people also tend to activate mental representations of God, which can be another form of attachment for many people (Granqvist, Mikulincer, Gewirtz, & Shaver, 2012). These experimental outcomes underscore our tendency to turn to trusted attachment figures when we are under threat.

## Social baseline theory

Another important theory to consider in relation to the role of attachment and trauma is social baseline theory (Beckes & Coan, 2011; Coan & Sbarra, 2015). It argues that social relationships play an important role in effectively minimising use of energy, which accords with notions that a fundamental driver of neural activity is to efficiently utilise energy resources (Proffitt, 2006). This theory posits that humans are hardwired to connect with each other as a result of evolutionary processes that have resulted in our brains being wired in such a way that the brain's default state is one in which it expects social attachments to be proximal. Social baseline theory proposes that a major function of social attachments has been to conserve energy, including basic functions as thermoregulation and limiting exposure to risk of predators (IJzerman et al., 2015). It is proposed that our strong reliance on caregivers and others in early years to provide warmth, protection, and nurture results in innate tendencies for proximity to others as a means of sharing the load of survival (e.g., sharing responsibilities with a partner), protecting us from threats (e.g., forming safety by turning to others), and keeping us warm. The issue of thermoregulation is fundamental to survival because many life-dependent functions rely on satisfactory body temperature, and to maintain this temperature requires considerable expenditure of energy. Accordingly, social baseline theory posits that social relationships are central for conserving our energy by keeping us warm, thereby not wasting energy on unnecessary tasks (IJzerman et al., 2015).

Inherent in social baseline theory is that the innate programming of social proximity that exists in many species influences processes at a very fundamental level. This is evident from cross-species research. For example, rodents housed in groups display reduced metabolic rates as group size increases (Nunez-Villegas, Bozinovic, & Sabat, 2014). Furthermore, rodents who huddle together have higher body temperature (Gilbert, McCafferty, Giroud, Ancel, & Blanc, 2012). The result of this thermoregulatory effect on the biosystem is that it allows for energy to be allocated to other functions necessary to survive in the face of threats. This pattern highlights that from a very early age, we are programmed at fundamental biological levels to seek proximity to others because they have been essential for our survival.

## Do attachments help people manage adversity?

Seeking proximity to social attachments is one of the core strategies utilised to cope with stressful experiences (Mikulincer & Shaver, 2012). Supporting the importance of attachments for management of adversity is evidence that the presence of social supports ameliorates fundamental stress responses at experiential and neural levels (Coan, Schaefer, & Davidson, 2006). Activating mental representations of attachment figures (e.g., by presenting an image of a mother holding a baby) leads to reduced attentional bias to threat (Mikulincer et al., 2002), positive endorsement of neutral stimuli (Mikulincer & Shaver, 2001), enhanced prosocial behaviour (Mikulincer, Shaver, Gillath, & Nitzberg, 2005), reduced pain perception (Master et al., 2009), reduced noradrenergic activation following a stressor (Bryant, & Chan, 2015), and diminished pain-related neural activation (Eisenberger, Jarcho, Lieberman, & Naliboff, 2006).

## Individual differences in attachment

Attachment theorists have noted that diminished attentive care during infancy can result in an insecure attachment relationship and possibly an inadequate internal working model of attachment to be utilised during later childhood and adulthood (Bowlby, 1961). This, in turn, is believed to result in the development of secondary attachment strategies, and potentially maladaptive or dysfunctional behaviours, emotions, and cognitions. Through a series of early studies, Ainsworth and colleagues noticed that these secondary attachment strategies arose when children's needs were responded to in an erratic and emotionally distant manner by their primary caregiver (Ainsworth, Boston, Bowlby, & Rosenbluth, 1956). Children would commonly either blunt or intensify their emotional expressions in order to maintain the relationship and maximise their caregiver's availability (Ainsworth, 1973). In this way, early life experiences give the foundation to a person's attachment style, which influences how people can subsequently use attachments; this in turn becomes a source of individual differences in emotion regulation across the lifespan (Mikulincer & Shaver, 2007a).

Attachment styles are typically conceptualised in two dimensions; attachment-related anxiety and attachment-related avoidance (Brennan & Shaver, 1998). Attachment-related anxiety is a dimension that reflects the extent to which an individual worries about the proximity and/or availability of his/her partner in times of need. The second dimension, attachment-related avoidance, reflects the extent of a person's distrust to others and to which an individual maintains behavioural independence and emotional distance from his/her partner to avoid abandonment. A person with high attachment–related anxiety is more likely to use *hyperactivating* strategies to attain proximity, support, and love from others. This can lead them to constantly seek proximity because previously such tactics were sometimes successful in making this person feel secure. Conversely, someone with attachment avoidance would use *deactivating* strategies that are highly self-reliant and typically distance oneself from others. Such a person typically avoids expressing his/her distress, anxiety, and despair to others in order to inhibit proximity seeking when coping with stress (Fraley & Shaver, 1997; Mikulincer, & Florian, 1995).

People with insecure attachments distrust partners (Hazan & Shaver, 1987), have lower self-esteem (Mickelson, Kessler, & Shaver, 1997), and are more likely to develop posttraumatic stress disorder (PTSD) following war exposure (Dieperink, Leskela, Thuras, & Engdahl, 2001). Furthermore, when threatened, individuals with insecure attachments have slower reaction times in recognising the names of their secure attachment figures (Mikulincer et al., 2002). People with avoidant attachment tendencies distance themselves during threat processing as a means of coping; supporting this proposal is evidence that during threat avoidantly attached individuals inhibit proximity-seeking behaviour and are less likely to activate attachment representations (Mikulincer, Birnbaum, Woddis, & Nachmias, 2000).

However, some commentators have proposed that there are distinct advantages in having an insecure attachment style (Belsky, 1999). Whereas secure attachment systems buffer people against vulnerability for psychiatric conditions (Mikulincer, & Shaver, 2007b) and enhance self-efficacy and capacity to cope with stressors (Mikulincer, & Florian, 1998), it is argued there needs to be an evolutionary rationale for why over one-third of people have an insecure attachment style (Ein-Dor, Mikulincer, Doron, & Shaver, 2010). It is posited that for the species to survive it is necessary for some people to have insecure attachment styles because these can promote safety at times of threat. For example, an anxiously attached person may be vigilant and detect threat before securely attached people who otherwise feel comfortable because they are supported by others; that is, they behave as the *sentinels* of the group who remain alert to potential danger. Additionally, avoidantly attached people who are focused on individual survival may develop means of escape that others can adopt. Put another way, securely attached individuals may be deprived at times of threat because their tendency to seek comfort in others may slow their detection of danger and also inhibit the fight/flight response (Ein-Dor et al., 2010). There is evidence to support these views. For example, anxiously attached individuals are more likely to interpret threatening situations in ways consistent with a sentinel role insofar as they detected threats and warned others; avoidantly attached individuals respond in ways consistent with a fight/flight response (Ein-Dor, Mikulincer, & Shaver, 2011). These findings suggest that insecure attachment is not uniformly disadvantageous and that there may be distinct benefits in coping with trauma for these particular individuals.

## Attachments and trauma

Relatively less empirical work has been conducted that directly tests how activating mental representations of attachments may impact recovery from trauma. Two interesting studies have emerged from Israel. One of the robust findings in PTSD research is that people with PTSD show greater interference on an emotional Stroop test (Bryant & Harvey, 1995; McNally, English, & Lipke, 1993). This test requires the participant to name the colour of the words that are printed in, and those with PTSD are slower to name the colour of the threat-related words (war, rape, etc.). In one Israeli study, students who had survived terrorist bombing attacks and had either elevated or low PTSD responses were administered by the emotional Stroop test; however, on each trial prior to the presentation of the words they were subliminally presented with an attachment-security word or a non-related word (Mikulincer, Shaver, & Horesh, 2006). This study replicated previous reports by finding that the provision of the attachment prime reduced the expected interference effect in participants with PTSD symptoms. In a replication study with prisoners of war from the Yom Kippur War, that used the same protocol, the beneficial effect of providing attachment primes was not observed (Mikulincer, Solomon, & Shaver, 2014). The authors concluded that the experience of being a prisoner of war may have damaged these individual's attachment systems to such an extent that they were not able to access internal attachment systems in a way that was helpful for them.

## Trauma and social support

In the context of considering the role of social processes in trauma response, it is worth noting that enormous attention has focused on the role of social support in PTSD and other posttraumatic reactions. Interestingly, the evidence concerning the beneficial effects of social support on PTSD is very mixed (Andrews, Brewin, & Rose, 2003; Borja, Callahan, & Long, 2006). There is evidence that positive social support is linked to improved later mental health, and negative social support is associated with poorer mental health (Grills-Taquechel, Littleton, & Axsom, 2011; Holeva, Tarrier, & Wells, 2001). Others have linked negative (but not positive) social support to subsequent posttraumatic distress (Andrews, Brewin, & Rose, 2003; Zoellner, Foa, & Brigidi, 1999) or found that the relationship between social support and PTSD symptoms changes over time (Cook & Bickman, 1990; Robinaugh et al., 2011).

One interpretation of the available evidence is the social support deterioration model, which holds that trauma may lead to disruptions in social support, which can be compounded by changes in people's expectations of social support, which in turn weakens interpersonal relationships (Barrera, 1988; Wheaton, 1985). In a longitudinal study, King and colleagues observed that more severe PTSD 2 years after combat was associated with lower positive social support 5 years later amongst male veterans (King, King, Taft, Hammond, & Stone, 2006). Interestingly, social support did not predict subsequent changes in PTSD symptoms. Although Kaniasty and Norris (1993) found that positive social support at 6 months predicted lower levels of PTSD 12 months following a natural disaster, between 12 and 18 months high levels of positive social support predicted decreases in PTSD and high levels of PTSD symptoms predicted decreases in social support. Taken together, there seems to be evidence that PTSD symptoms are associated with subsequent decreases in positive social support. Considering the potential benefits that social attachments can confer on people, this detrimental impact of PTSD on social support may serve to compound trauma survivors’ difficulties.

## A societal perspective

One potential criticism of prior research into social processes impacting trauma response is that the PTSD field has adopted a predominantly “egocentric” perspective in which individuals are asked about their social networks, and inferences are drawn from this information. This approach contrasts with developments in other fields of study, including sociology, economics, anthropology, mathematics, political science, and social psychology that have adopted a “sociocentric” approach that maps the interactive impacts of individuals and groups on societal levels (Christakis & Fowler, 2009). An approach termed social network analysis examines both social structures and individual attributes of people within these social structures—thereby allowing mapping of how these individual characteristics may be influenced by, and have an influence upon, the individual's social networks (Christakis & Fowler, 2013).

Understanding how posttraumatic stress responses occur within a social network context is important because it can shed light on numerous core mechanisms that impact adjustment. First, a person's structural position within a network (e.g., they may be isolated, connected with only a few other people, or possibly strongly connected to many people) has been shown to be associated with a range of relevant characteristics. Typifying this possibility is evidence that having friends who are not friends with each other is predictive of suicidality in women (Bearman & Moody, 2004). Furthermore, one's social network structure can impact on how emotional and behavioural features may impact people within the network. For example, posttraumatic mental health in one person may contribute to the mental health of others via a contagion effect, people with PTSD may selectively choose others with PTSD to socialise with, or people with PTSD may become connected because they engage in common activities (e.g., excessive alcohol abuse) (Valente, 2005). Although social network analyses have not been applied specifically to the study of posttraumatic mental health, it has been used to shed light on social determinants of a range of mental and general health functions. This work has noted that where one is positioned within a social network, and the connectedness of that social network, moderates many general health outcomes, including obesity (Christakis & Fowler, 2007), tobacco use (Christakis & Fowler, 2008), and alcohol consumption (Rosenquist, Murabito, Fowler, & Christakis, 2010). Furthermore, the nature of one's social networks impact on psychological functions, including depression (Rosenquist, Fowler, & Christakis, 2011), happiness (Fowler & Christakis, 2008), and loneliness (Cacioppo, Fowler, & Christakis, 2009). For example, one large population study found a contagion effect of depression especially in females, such that one's depression was associated with worsened depression in others in her network (Rosenquist et al., 2011).

To address this issue in the aftermath of trauma, a longitudinal study in Australia has commenced following a very large-scale natural disaster (Gibbs et al., 2013). The “Black Saturday” fires of 7 February 2009 in Victoria, Australia, led to the loss of life and much damage to physical infrastructure, resulting in 173 fatalities, 450,000 ha burned, 40 townships affected, 3,500 buildings damaged or destroyed, and a significant upheaval on the social infrastructure within communities. As this disaster affected small country towns and villages, it lent itself to a social network analysis because many people potentially knew others who were affected by the disaster. In addition to conducting a standard post-disaster psychiatric epidemiological survey, this study has asked all respondents to nominate up to 10 people who provide them support, and to whom they give support. Detailed information was obtained on all participants so that matching of named individuals across respondents can be achieved. This approach will allow this study to map the structures of social networks after the disaster and during the recovery phase, as well as understanding how posttraumatic mental health patterns impact on people who are socially connected.

## The challenge for the trauma field

It is time that the study of traumatic stress embraced more socially informed paradigms. Although there is convergent agreement that trauma does not occur in a social vacuum and we need to appreciate the role of interpersonal and societal factors, our paradigms have not addressed this need sufficiently. The social network approach provides a framework, which can be implemented following traumatic events, that allows us to understand how posttraumatic symptoms impact on social behaviours and vice versa. Longitudinal designs are needed to map these relationships, and this is underscored by findings from multi-wave studies that have shown the causal contribution of social processes on mental health (Christakis & Fowler, 2013). This is especially important in the aftermath of traumatic events that have direct societal impacts, such as large-scale disasters. Events such as earthquakes, hurricanes, and terrorist attacks can cause marked social upheaval, and how the individual functions within such social networks may be critical to adaptation.

The considerable experimental and clinical work conducted on the potential benefits of social attachments underscores the importance of fostering social connections. It is a concern that trauma, especially prolonged and interpersonal trauma, can negatively impact attachments. It appears that suffering prolonged trauma may impair one's attachment system, and thereby hinder one's capacity to benefit from attachments (Mikulincer, Solomon, & Shaver, 2014). This raises a significant challenge for understanding to better enhance trauma recovery and even augment treatment response. Neuroscience is also providing new insights into how biological processes implicated in attachments may impact on emotion processing, and possibly on facilitating treatment of PTSD. For example, the neuropeptide oxytocin, which has been shown to facilitate bonding in securely attached people, can effectively limit PTSD if provided in the acute period after trauma (Olff et al., 2014). If providing attachment can offer psychological benefit then it can provide a potentially useful means to assist trauma recovery. However, if some traumatised individuals are less able to access these attachment systems as a result of prior adverse attachment experiences, then we need to develop more targeted approaches to either foster attachment capability or develop non-attachment strategies that can also be beneficial.

The accumulating evidence suggests that how we function in our social world after trauma is a highly complex issue. Individual differences in how we seek out and benefit from attachments impact on the actual availability of social connections, which in turn can markedly impact on how we think, feel, and behave. The field of psychotraumatology has traditionally studied these issues from the perspective of an individual but lessons learned from other disciplines are informing us that a fuller understanding requires broader methodologies that recognise the interactive impact of the social networks in which people recover from traumatic events. The many empirical advances made in studies of attachment need to be extended to survivors of trauma, where there is a disappointing lack of empirical research related to attachment. On the basis of the work reviewed here, we would expect that those with secure attachment styles would benefit from having social supports in the aftermath of trauma. In contrast, those with avoidant attachment tendencies may engage other secondary coping strategies to manage their experience. Little work has been done to articulate these strategies and there is a need to develop a better understanding of how those with insecure attachments deal with trauma, and the extent to which these different strategies are adaptive. The accumulating evidence suggests that social support will be variably helpful to people after trauma, depending on one's attachment style; however, there is a need to establish an evidence base on how insecurely attached people are to be optimally helped in the aftermath of trauma, including the use of different treatment strategies.
